# Pneumococcal Pyopericardium With Xiphoid Osteomyelitis in HIV/AIDS: A Rare but Potentially Fatal Complication

**DOI:** 10.1155/cric/5008190

**Published:** 2025-08-13

**Authors:** Nihar Jena, Izza Saeed, Mackenzie Hamilton, Christian Tocquica, Sreekant Avula, Ramiz Sayyed, Juan Bastidas, Mark Studeny

**Affiliations:** ^1^Department of Interventional Cardiology, Marshall University, Huntington, West Virginia, USA; ^2^Department of Cardiology, Trinity Health Oakland/Wayne State University, Pontiac, Michigan, USA; ^3^Department of Diabetes, Endocrinology and Metabolism, University of Minnesota, Minneapolis, Minnesota, USA; ^4^Department of Cardiovascular Surgery, Marshall University, Huntington, West Virginia, USA

**Keywords:** HIV, pericardial tamponade, pyopericardium, *Streptococcus pneumoniae*

## Abstract

**Background:** Purulent pericarditis is a rare, life-threatening condition that has become exceedingly uncommon due to widespread use of antibiotics. However, immunocompromised patients remain susceptible to such opportunistic infections. It is typically caused by direct extension or hematogenous spread from a secondary bacterial source.

**Case Description:** We report a 55-year-old man with HIV cardiomyopathy who was transferred from an outside facility for chest pain and dyspnea, with an electrocardiogram suggestive of an ST-segment elevation myocardial infarction, prompting emergent cardiac catheterization, which revealed normal coronary arteries. A bedside echocardiogram revealed a large pericardial effusion with tamponade physiology, leading to pericardiocentesis. The pericardial fluid analysis revealed a high white blood cell count and a significantly elevated lactate dehydrogenase level, and cultures were positive for *Streptococcus pneumoniae*. Despite receiving antibiotics, the effusion reaccumulated, necessitating a subxiphoid pericardial wash and drainage. The xiphoid biopsy was consistent with acute osteomyelitis. The patient was discharged with long-term antibiotics and scheduled outpatient follow-ups.

**Conclusion:** This case illustrates an unusual presentation of HIV-AIDS, highlighting advancements in managing pneumococcal pyopericardium with tamponade and the ongoing risk of opportunistic infections despite antiretroviral treatment. Though rare, pyopericardium can be fatal; so prompt diagnosis and multidisciplinary management are essential to improve outcomes.

**Summary**

• Immunocompromised patients are prone to develop pyopericardium. Maintaining a high index of clinical suspicion, a broader differential diagnosis, and utilizing appropriate diagnostic modalities like bedside echocardiography can prevent potential delays in diagnosis.
• A multispecialty approach involving cardiologists, cardiac surgeons, and infectious disease specialists is recommended for the holistic management of complex infective pericardial conditions.
• Long-term follow-up is recommended to identify potential late complications, including recurrence, constrictive pericarditis, or progression of underlying systemic disease.

## 1. Introduction

Pyopericardium is a rare condition accounting for < 1% of causes of pericarditis, with a high mortality rate of 40% in treated patients [1]. In untreated patients, the mortality is 100% in which infection propagates in the pericardial space, leading to a pus-filled pericardial effusion and cardiac tamponade, which can cause cardiogenic shock and death [2]. In the current era of antibiotic resistance, we may see a resurgence of this rare infective condition, especially in immunocompromised populations such as patients with human immunodeficiency virus–acquired immune deficiency syndrome (HIV-AIDS) [3]. Therefore, it is imperative to formulate the correct diagnosis and implement timely management. The case here depicts a 55-year-old on antiretroviral therapy (ART) who developed pyopericardium from an uncommon pathogen, *Streptococcus pneumoniae* (SP). A multidisciplinary approach was adopted to manage this complex life-threatening condition.

## 2. Case Presentation

The patient is a 55-year-old male with a past medical history of Type 2 diabetes mellitus, hypertension, HIV compliant with combination ART bictegravir, emtricitabine, and tenofovir alafenamide (Biktarvy), and recently diagnosed nonischemic cardiomyopathy with left ventricular ejection fraction (LVEF) of 27%, who presented to an outside facility with complaints of chest pain and dyspnea for the past day. The patient reported nausea and vomiting over the past 3 days, body aches, chills, diaphoresis, and low urine output. He also reports a worsening cough with the production of greenish sputum over the past week. He has been compliant with his ART. Notably, the patient had been recently treated for influenza. On physical examination, he was diaphoretic and was in significant distress—heart rate: 131 bpm, blood pressure: 115/91 mmHg, respiratory rate: 33 breaths/min, oxygen saturation: 95% on 3-L nasal cannula oxygen, afebrile. Chest auscultation was significant for bilateral decreased breath sound with crepitation; cardiac auscultation was significant for dull heart sound, no murmur or rub.

## 3. Investigations

**Figure 1:**
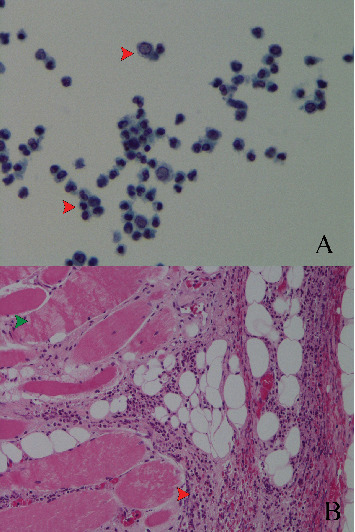
(A) Pericardial fluid: rare benign mesothelial cells and numerous acute inflammatory cells (red arrow) (cytology, Papanicolaou stain, 600× magnification). (B) Pericardium, biopsy: benign fibroconnective tissue, muscle cells (green arrow) with acute inflammatory cells (red arrow) (histology, H&E stain, 200× magnification).

The patient's laboratory analysis revealed a CD4 count of 254 cells/*μ*L with an HIV viral load of 32 copies/mL. Inflammatory markers were elevated with leukocytosis (34.5 × 10^3^ cells/*μ*L) and thrombocytosis (470 × 10^3^ cells/*μ*L). Renal function was significantly impaired with a creatinine of 4.75 mg/dL. Cardiac biomarkers were notable for troponin elevations ranging from 28 to 34 ng/L and a B-type natriuretic peptide (BNP) level of 550 pg/mL. Blood and xyphoid bone culture were positive for SP. The pericardial fluid analysis revealed a turbid, green appearance with an elevated white blood cell (WBC) count of 188,771/*μ*L, predominantly neutrophilic (92%), along with a lactate dehydrogenase (LDH) level of 18,125 U/L, a total protein level of 6.2 g/dL, an albumin level of 2.8 g/dL, and a glucose level of 241 mg/dL. Cytology revealed inflammatory cells (Figure 1A). The pericardial fluid culture also grew SP, confirming the presence of bacterial pericarditis (Table 1).

**Figure 2:**
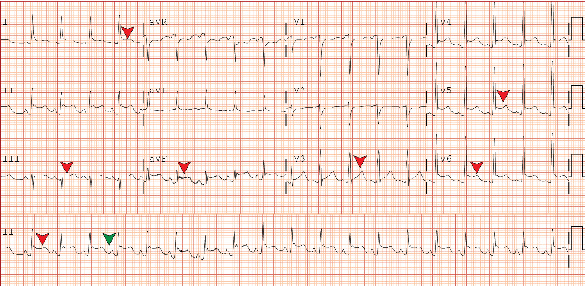
Electrocardiogram showing diffuse ST segment elevation (red arrow) and PR depression (green arrow).

**Figure 3:**
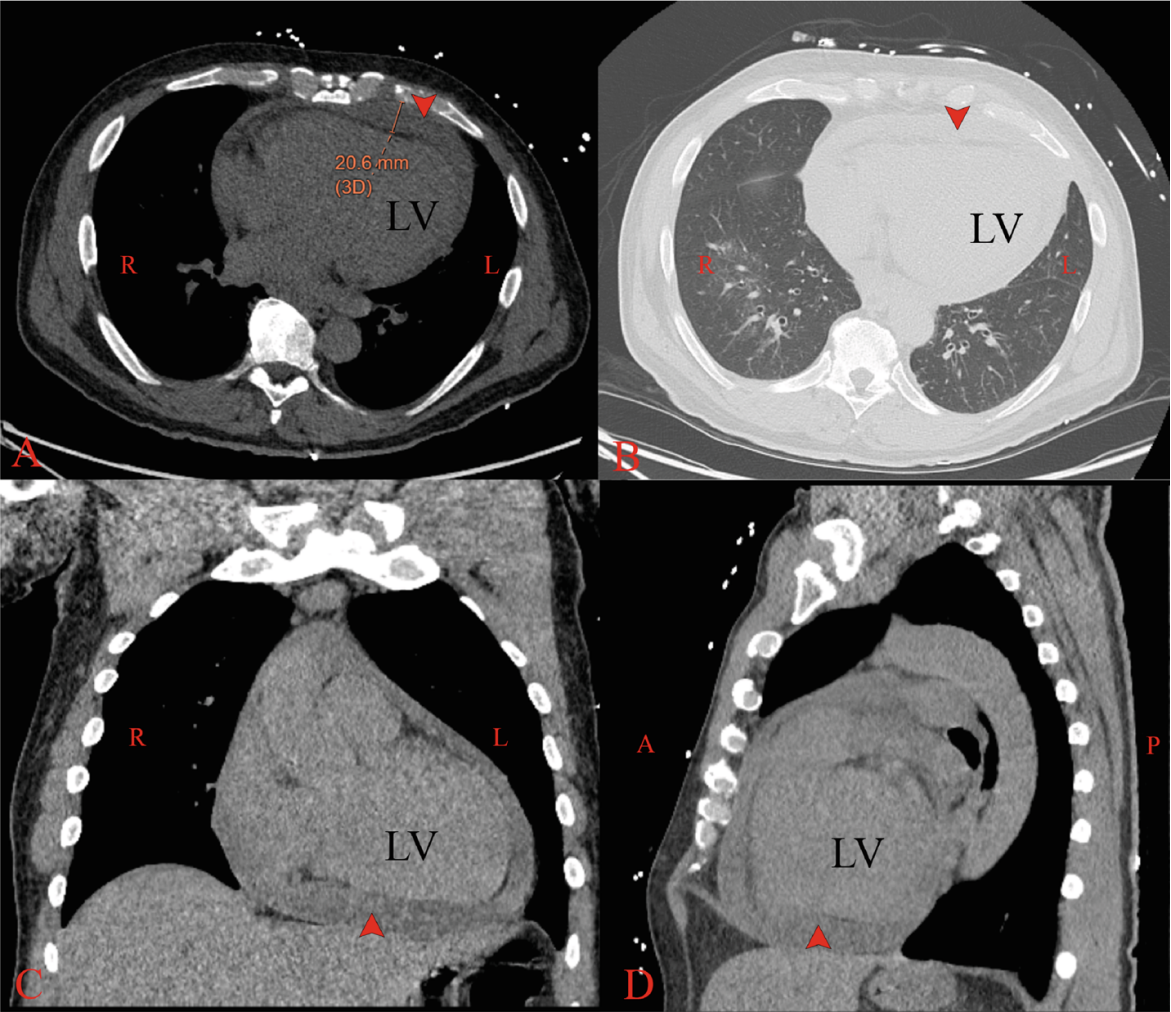
(A–D) Thoracic computed tomography showing pericardial effusion (red arrow). A: anterior, P: posterior, R: right, L: left, LV: left ventricle.

**Figure 4:**
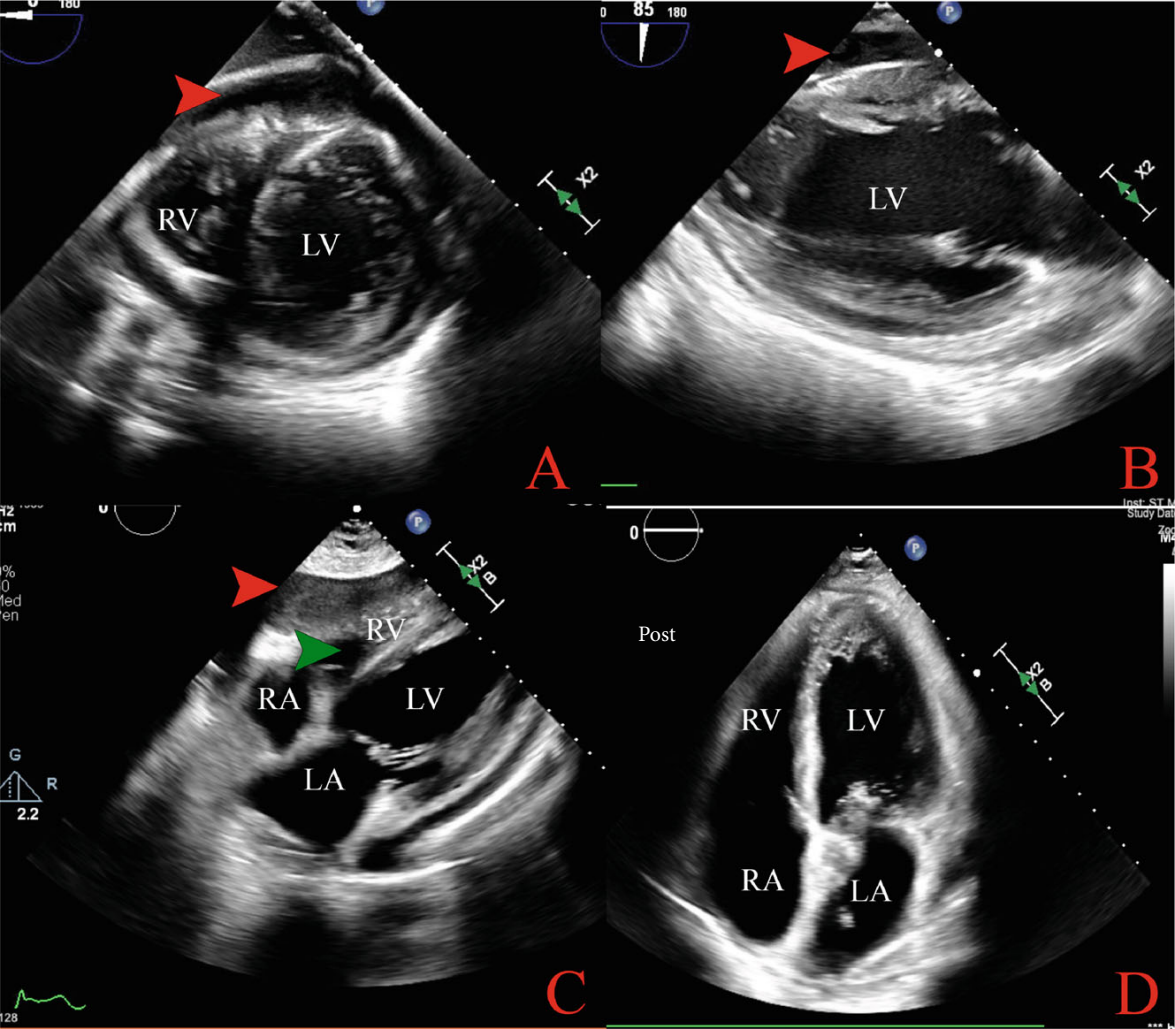
Echocardiogram. (A) Parasternal short-axis view showing large pericardial effusion (red arrow). (B) Parasternal short-axis view showing pericardial effusion (red arrows). (C) Parasternal short-axis view showing pericardial effusion (red arrow), evidence of RV collapse suggesting cardiac tamponade (green arrow). (D) Postpericardiocentesis apical four-chamber showing drained pericardial fluid. RA: right atrium, RV: right ventricle, LA: left atrium, LV: left ventricle.

**Figure 5:**
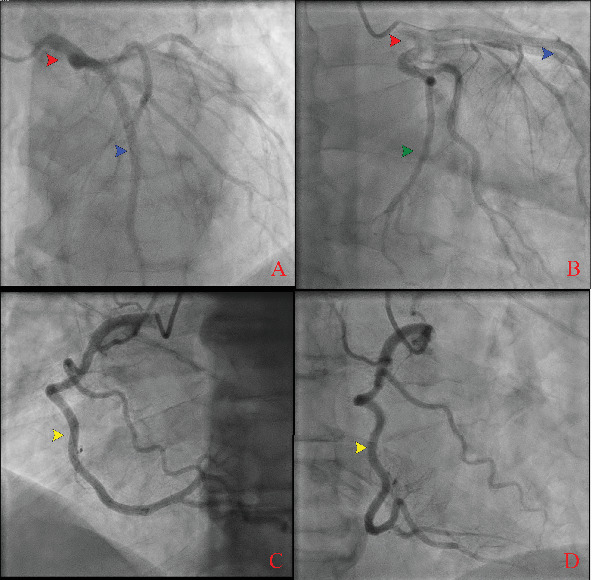
(A–D) Coronary angiogram showing normal epicardial coronary arteries: left main coronary artery (red arrow), left anterior descending artery (blue arrow), left circumflex artery (green arrow), and right coronary artery (yellow arrow).

Electrocardiography (EKG) showed sinus tachycardia with inferolateral ST-segment elevations, PR depression, and T-wave abnormalities (Figure 2). A chest radiograph revealed mild cardiac enlargement, but no acute intrathoracic pathology was noted. Computed tomography of the chest showed bilateral diffuse centrilobular micronodules with mild patchy ground-glass opacities, as well as a large pericardial effusion measuring 2.1 cm anterior to the right ventricle, with fibrin strands present (Figure 3). Transthoracic echocardiography (TTE) showed a reduced LVEF of 25% with a large circumferential pericardial effusion measuring 2.5 cm, consistent with impending tamponade (Figure 4). Left heart catheterization did not demonstrate any significant angiographic coronary artery disease (Figure 5) (Table 2).

## 4. Management

**Figure 6:**
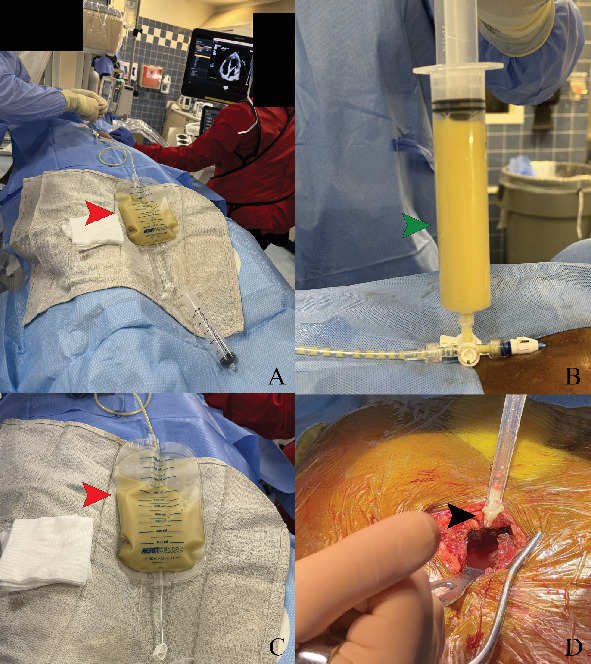
(A–C) Cardiac Cath Lab echocardiographic and fluoroscopic-guided pericardiocentesis showing purulent pericardial fluid aspiration (red arrow and green arrow). (D) Surgical pericardial fluid drainage and washout (black arrow showing thick purulent materials).

He was taken to the cardiac Cath Lab and underwent pericardiocentesis. Initially, the patient had about 775 mL of purulent fluid drained. The pericardial drain was left in place. It was removed after 48 h when drainage was minimal. Since the drain placement, the patient has had 1.7 L of fluid drained. However, the next day, a repeat echo showed reaccumulation of fluid. Cardiothoracic surgery was consulted, and the patient underwent subxiphoid drainage and washout of the pericardium (Figure 6). Two chest tubes were secured in place over the next 3 days. A total of 400 mL of fluid was drained during this time. They were removed from the drains when there was less than 50 cc output over 24 h. Infectious diseases were also consulted, and the patient was initially treated with broad-spectrum antibiotics. Once blood cultures revealed SP to be sensitive to ceftriaxone, antibiotic therapy was tailored to IV ceftriaxone 2 g every 24 h, to be administered over a 14-day course from the date of surgery.

## 5. Outcome and Follow-Up

On Day 11, the patient was discharged in stable condition with long-term antibiotics. The histopathology report of the pericardial tissue revealed no evidence of malignancy, instead showing benign fibroconnective tissue and skeletal muscle with acute inflammation. Pericardial fluid analysis showed rare benign mesothelial cells and numerous acute inflammatory cells (Figure 1B). The patient had an outpatient follow-up with TTE, which showed a trivial pericardial effusion.

## 6. Discussion

HIV/AIDS has long been recognized as a condition that predisposes individuals to opportunistic infections. However, with the advent of ART, the incidence of pericardial effusion has been declining [4]. Asymptomatic pericardial effusion is generally considered a benign condition in HIV patients. Nevertheless, the occurrence of pericardial effusion in individuals undergoing ART is quite rare. In a study by Lind et al., only 0.25% of patients on ART experienced pericardial effusion [5]. Overall, in the developed world, pyopericardium, or purulent pericarditis, is very uncommon, accounting for less than 1% of all pericarditis cases. If left untreated, it carries a mortality rate close to 100% due to the risk of cardiogenic shock and cardiac tamponade [6].

The diagnosis of pericardial tamponade is primarily clinical; however, TTE confirmation and pericardial fluid aspiration are essential for diagnosing pyopericardium. The usual presentation is fever, chest pain, congestive heart failure, and later sepsis and cardiogenic shock. Central venous pressure is often elevated, while blood pressure tends to be low. Acute coronary syndrome (ACS) or other conditions may mimic symptoms of this condition, making the diagnosis challenging. In our case, the patient was initially treated as an ST-elevation myocardial infarction similar to a case reported by Smith et al. [7]. The patient was referred from an outside facility and was taken directly to the cardiac catheterization laboratory for emergent cardiac catheterization, following the standard ACS protocol due to reported ST elevations on the EKG. However, in retrospect, considering the classical EKG findings that indicated signs of pericarditis, it would have been ideal to perform a bedside TTE promptly, as this could have diagnosed pericardial effusion earlier. This situation highlights an important lesson about the necessity of keeping a broader differential diagnosis in immunocompromised patients, where symptoms can often be overlapping.

*Staphylococcus* and *Streptococcus* species are the most common pathogens responsible for pyopericardium. However, Gram-negative bacteria and fungi are also increasing in prevalence among immunocompromised patients. Common sources of pyopericardium include pneumonia, empyema, thoracic surgery, and hematogenous spread associated with sepsis. Rare causes include rupture of perivalvular abscesses in endocarditis and spread from the oral cavity along fascial planes. Factors that increase the risk of pericarditis include immunosuppression, alcohol abuse, and prior pericardial inflammation and fibrosis.

SP is a rare cause in the postantibiotic era. However, HIV-positive individuals, particularly those with advanced immunosuppression (CD4 < 200 cells/*μ*L), have a significantly higher risk of invasive pneumococcal disease, including pericarditis. The patient, in this case, developed pyopericardium despite being on ART with a CD4 count well beyond the threshold of pneumococcal infection. Several hypotheses can explain the paradox. First, despite the adequacy of CD4 counts and viral suppression, there is literature suggesting a lack of qualitative antibody-mediated response for bacterial opsonization in HIV patients, as explained by the B cell exhaustion theory [8]. Secondly, a recent influenza infection might have disrupted the respiratory epithelial barriers, predisposing to pneumococcal invasion and leading to pyopericardium. Also, there are recent studies highlighting coinfection of influenza with pneumococcal, showing significantly higher morbidity in otherwise controlled HIV patients [9]. The synergetic pathogenic mechanism could elucidate the outcome observed in our patient, emphasizing the need for careful monitoring and complete treatment of this subgroup of immunocompromised individuals.

Treatment involves draining the pyopericardium, administering antibiotics, and performing a pericardiectomy. Potential complications of pericardial catheter insertion include pneumothorax and cardiac or coronary laceration, which can be minimized with echocardiographic guidance. The pericardial catheter is typically left in place to facilitate further drainage and prevent the recurrence of effusion, which can occur in up to 75% of cases [2]. It is recommended that the catheter be removed once the drainage is less than 25 mL per day. Some literature also suggests an epicardiectomy to allow for improved movement of the myocardium, with the “turtle cage” technique proposed as an alternative, where slits are made in the epicardium to release fibrotic areas. A similar case was reported by Cracknell et al.; a 52-year-old female patient underwent complete pericardiectomy along with prolonged antibiotics [10]. The long-term goal is to prevent recurrence and constrictive pericarditis. In our patient's case, epicardial irrigation and debridement were performed successfully.

## 7. Conclusion

Maintaining a high clinical suspicion and ensuring prompt diagnosis of pyopericardium can significantly impact patient outcomes. Although uncommon, immunocompromised patients should be carefully evaluated for this condition. Engaging in a multidisciplinary discussion and involving patients in shared decision-making are essential to establish clear expectations regarding management plans and potential outcomes, particularly given the high morbidity and mortality rates.

## Figures and Tables

**Table 1:** Biochemical and microbiology laboratory results.

**Parameter**	**Value**	**Normal reference range**	**Unit**
CD4 count	254	500–1500	Cells/*μ*L
HIV viral load	32	Undetectable	Copies/mL
WBC	34.5	4–11	×10^3^/*μ*L
Platelets	470	150–400	×10^3^/*μ*L
INR	4.0	0.8–1.2	
Sodium	122	135–145	mmol/L
Potassium	4.3	3.5–5.2	mmol/L
Chloride	83	95–105	mmol/L
Bicarbonate	18	22–28	mmol/L
Anion gap	21	8–16	
BUN	95	7–20	mg/dL
Creatinine	4.75	0.6–1.2	mg/dL
Glucose	495	70–140	mg/dL
Procalcitonin	7.96	< 0.15	ng/mL
CRP	45.1	< 10	mg/L
ESR	22	0–20	mm/h
Troponin (peak)	34	< 0.04	ng/L
BNP	550	< 100	pg/mL
Lactic acid	3.9	< 2.0	mmol/L
Pericardial fluid WBC	188,771	< 5000	/*μ*L
Pericardial fluid LDH	18,125	< 200	U/L
Pericardial fluid protein	6.2	6–8	g/dL
Pericardial fluid glucose	241	40–80	mg/dL
Pericardial fluid culture	*Streptococcus pneumoniae* (+)	Negative	
Blood culture	*Streptococcus pneumoniae* (+)	Negative	
Respiratory panel	Coronavirus 229E-HKU1-NL63-OC43 (+)	Negative	
Urine *Streptococcus pneumoniae* antigen	Positive	Negative	

**Table 2:** Imaging results.

**Investigation**	**Findings**
Electrocardiogram	Sinus tachycardia, inferolateral ST elevations, PR depression
Chest x-ray	Mild cardiac enlargement, no acute process
Chest computed tomography	Centrilobular micronodules, ground-glass opacities, 2.1-cm pericardial effusion
Transthoracic echocardiogram	Ejection fraction 25%, large effusion (2.5 cm), impending tamponade
Coronary angiogram	No significant coronary artery disease

## Data Availability

Data sharing does not apply to this article as no new data were created or analyzed in this study.

## References

[1] Maisch B., Seferović P. M., Ristić A. D. (2004). Guidelines on the Diagnosis and Management of Pericardial Diseases Executive Summary: The Task Force on the Diagnosis and Management of Pericardial Diseases of the European Society of Cardiology. *European Heart Journal*.

[2] Sagristà-Sauleda J., Barrabés J. A., Permanyer-Miralda G., Soler-Soler J. (1993). Purulent Pericarditis: Review of a 20-Year Experience in a General Hospital. *Journal of the American College of Cardiology*.

[3] Mandell L. A., Wunderink R. G., Anzueto A. (2007). Infectious Diseases Society of America/American Thoracic Society Consensus Guidelines on the Management of Community-Acquired Pneumonia in Adults. *Clinical Infectious Diseases*.

[4] Heffernan R. T., Barrett N. L., Gallagher K. M. (2005). Declining Incidence of Invasive *Streptococcus pneumoniae* Infections Among Persons With AIDS in an Era of Highly Active Antiretroviral Therapy, 1995–2000. *Journal of Infectious Diseases*.

[5] Lind A., Reinsch N., Neuhaus K. (2011). Pericardial Effusion of HIV-Infected Patients - Results of a Prospective Multicenter Cohort Study in the Era of Antiretroviral Therapy. *European Journal of Medical Research*.

[6] Bhaduri-McIntosh S., Prasad M., Moltedo J., Vázquez M. (2006). Purulent Pericarditis Caused by Group A Streptococcus. *Texas Heart Institute Journal*.

[7] Smith Z. A., Wimbush S. (2012). Pneumococcal Pyopericardium Masquerading as Acute Coronary Syndrome. *Journal of the Intensive Care Society*.

[8] Moir S., Fauci A. S. (2014). B-Cell Exhaustion in HIV infection. *Current Opinion in HIV and AIDS*.

[9] McCullers J. A. (2006). Insights Into the Interaction Between Influenza Virus and Pneumococcus. *Clinical Microbiology Reviews*.

[10] Cracknell B. R. S., Ail D. (2015). The Unmasking of a Pyopericardium. *BMJ Case Reports*.

